# The Effect of Verbal Working Memory Intervention on the Reading Performance of Students with Specific Learning Disabilities

**DOI:** 10.3390/bs15030356

**Published:** 2025-03-13

**Authors:** Mehmet Okur, Veysel Aksoy

**Affiliations:** 1 Dede Korkut Faculty of Education, Department of Special Education, Kafkas University, 36000 Kars, Turkey; 2 Research Institute for Individuals with Disabilities, Anadolu University, 26000 Eskisehir, Turkey; vaksoy@anadolu.edu.tr

**Keywords:** verbal working memory, specific learning disabilities, reading intervention, reading comprehension

## Abstract

The purpose of this study is to investigate the effects of verbal working memory (VWM) interventions on reading speed, accuracy, and comprehension in elementary school students diagnosed with specific learning disabilities (SLD). Given the limited research on the role of VWM in reading performance, this study fills a critical gap in the literature. A pre-test and post-test design was employed, with an experimental group *(n* = 14) receiving VWM interventions over 4 weeks, while the control group (*n* = 12) received no intervention. The intervention focused on enhancing VWM and verbal short-term memory (V-STM) through structured cognitive tasks, including rehearsal techniques and phonological loop strengthening activities, delivered over 24 sessions. Results showed that although VWM interventions significantly enhanced VWM capacity (*t*(24) = 3.39, *p* < 0.05, *d* = 1.48), they did not lead to significant improvements in reading speed or accuracy. However, a statistically significant improvement in reading comprehension was observed (*p* = 0.04, *d* = 0.92). These findings suggest that while enhancing VWM may not directly improve reading fluency, it can positively affect comprehension. The study highlights the importance of considering VWM in educational interventions targeting reading comprehension and recommends further research into other cognitive and linguistic factors influencing reading speed and accuracy. Additionally, future studies should explore the long-term effects of diverse intervention strategies on reading outcomes.

## 1. Specific Learning Disability and Reading Problems

Specific Learning Disability (SLD) is a neurodevelopmental disorder that significantly disrupts the acquisition and utilization of core academic skills, including reading, writing, and mathematics. Despite individuals often demonstrating average or above-average intelligence, these challenges create a notable disparity between their intellectual potential and academic performance (American Psychiatric Association, 2013). These difficulties are not the result of a lack of effort or motivation but stem from underlying neurological impairments that affect specific domains of learning (Fletcher et al., 2019). These persistent challenges have led to the development of diverse intervention strategies aimed at supporting students with SLD. These interventions can be broadly categorized into behavioral and socio-emotional interventions (Torgesen, 2005; Wanzek et al., 2013), as well as cognitive interventions (Swanson & Hoskyn, 2001). Among these approaches, cognitive interventions have gained increasing attention due to their potential to directly target the cognitive deficits underlying reading difficulties (Swanson & Hoskyn, 2001; Melby-Lervåg & Hulme, 2013; Gathercole & Alloway, 2008).

Cognitive interventions specifically aim to enhance the cognitive mechanisms essential for successful reading development, including VWM, attention regulation, and executive functions. These interventions are grounded in well-established theoretical frameworks, such as Baddeley’s Working Memory Model (Baddeley, 2000). Compared to behavioral interventions that focus primarily on external behavioral reinforcement (Therrien, 2004) and socio-emotional interventions that target motivation and self-efficacy (Wigfield & Guthrie, 1997), cognitive interventions directly address the internal processes that govern reading performance. This makes them uniquely suited for addressing the root causes of reading difficulties in students with SLD.

VWM plays a central role within cognitive interventions, serving as a key mechanism that supports decoding, fluency, and comprehension processes. As a cognitive bottleneck, VWM governs the efficient coordination between decoding processes and higher-order comprehension skills, ensuring that linguistic input is continuously processed and integrated into coherent meaning (Baddeley, 2000; Cain et al., 2004). Consequently, cognitive interventions targeting VWM are expected to produce improvements in multiple dimensions of reading performance (Swanson & O’Connor, 2009).

Reading difficulties represent one of the most pervasive academic challenges among students with SLD, as successful reading development relies heavily on cognitive processes governed by VWM (Swanson & O’Connor, 2009; Cain et al., 2004; Gathercole & Alloway, 2008). Reading difficulties represent one of the most pervasive academic challenges among students with SLD, affecting their ability to engage effectively with academic content across various subjects (Snowling & Hulme, 2011). While deficits in decoding and fluency are well-documented barriers to comprehension, recent research emphasizes that these lower-level reading processes are strongly mediated by VWM, which governs the temporary storage, processing, and integration of linguistic information during reading (Swanson & O’Connor, 2009; Cain et al., 2004; Gathercole & Alloway, 2008). When students with SLD experience weak VWM capacity, they struggle to efficiently manage decoding and fluency demands, leaving limited cognitive resources available for higher-order comprehension processes, ultimately restricting their academic progress (Vellutino et al., 2004).

Although VWM has been widely recognized as a critical cognitive process supporting reading comprehension, empirical findings remain inconsistent regarding its specific contributions to different reading components, particularly reading speed, reading accuracy, and comprehension accuracy (Swanson & O’Connor, 2009; Cain & Oakhill, 2006). While some studies indicate that enhancing VWM capacity directly improves reading fluency and reduces reading errors (Swanson & O’Connor, 2009; Cain & Oakhill, 2006), others argue that VWM’s primary role lies in higher-order comprehension processes, with minimal direct influence on decoding and speed (Gathercole & Baddeley, 1993). Further complicating the picture, studies investigating the effects of VWM training present mixed results, with some reporting indirect benefits for fluency and accuracy (Swanson et al., 2006), while others find no measurable impact (Berninger et al., 2010).

These inconsistencies highlight a critical gap in the literature, particularly concerning the multidimensional effects of targeted VWM training on core reading components in students with SLD. This study aims to address this gap by systematically examining the simultaneous effects of VWM training on reading speed, reading errors, and reading comprehension in students with SLD, providing a comprehensive analysis of VWM’s role across multiple reading processes.

Extensive research highlights that word decoding and reading fluency represent two foundational processes that significantly contribute to reading comprehension among students with SLD (Ehri, 2005; Wawire & Zuilkowski, 2021). Word decoding refers to the process of translating written symbols into corresponding sounds, which enables word recognition and basic reading accuracy (Silverman et al., 2021). Reading fluency, defined as the ability to read with speed, accuracy, and appropriate prosody, is equally critical for enabling readers to focus on higher-order comprehension tasks (Hudson et al., 2005). However, these skills do not operate in isolation; VWM plays a central role in supporting both processes by enabling the temporary storage, manipulation, and integration of linguistic information during reading (Swanson & O’Connor, 2009; Cain et al., 2004).

Deficits in VWM exacerbate decoding and fluency difficulties, requiring students to devote excessive cognitive resources to lower-order processes, leaving insufficient capacity for meaning construction and inference generation (Gathercole & Alloway, 2008). Consequently, students with SLD often experience fragmented reading comprehension, struggling not only with basic accuracy and fluency but also with higher-level text integration skills (Vellutino et al., 2004). Although numerous studies have explored interventions targeting decoding and fluency directly (Therrien, 2004; Wanzek et al., 2013), more recent research highlights that cognitive interventions targeting VWM may offer an indirect but essential pathway to strengthening these foundational skills while also enhancing comprehension (Swanson & O’Connor, 2009; Melby-Lervåg & Hulme, 2013).

In addition to well-documented challenges in decoding and fluency, recent research highlights that students with SLD often process texts only at a superficial level, limiting their ability to construct meaning, establish coherence across ideas, and grasp the author’s intent (Rochman, 2018). This fragmented processing prevents students from fully engaging with the higher-order cognitive processes essential for deep comprehension, such as inference-making, evaluation, and critical analysis. Furthermore, deficits in prosodic reading—the ability to read with appropriate rhythm, intonation, and stress—further compound these difficulties (Pikulski & Chard, 2005). Weak prosody disrupts not only expressive reading but also the interpretation of emotional and semantic cues, making it more difficult for students to contextualize meaning (Veenendaal et al., 2015). These unresolved challenges limit students’ ability to access complex academic texts, ultimately restricting their academic development across content areas. Slower reading rates among students with SLD stem largely from the excessive cognitive resources required for word recognition and decoding, which limits their ability to efficiently construct meaning (Rochman, 2018). This cognitive overload—amplified by underlying weaknesses in VWM—prevents students from shifting their focus to higher-order comprehension processes, such as inference-making and evaluating textual coherence (Swanson & O’Connor, 2009; Gathercole & Alloway, 2008). Deficits in prosodic reading, which reflects the ability to apply rhythm, stress, and intonation, further contribute to fragmented comprehension by disrupting the extraction of semantic and emotional cues embedded in texts (Veenendaal et al., 2015; Miller & Schwanenflugel, 2008).

To address these multifaceted challenges, the literature offers a range of intervention strategies, broadly classified into fluency-based approaches, decoding accuracy interventions, and cognitive-based programs targeting underlying processing deficits (Torgesen, 2005; Wanzek et al., 2013). While direct instruction methods focus on strengthening decoding and fluency skills through repeated reading and phonological training (Hudson et al., 2005), cognitive-based interventions, particularly those targeting VWM, aim to enhance the cognitive capacity required to efficiently coordinate lower- and higher-order reading processes (Swanson & O’Connor, 2009). This study builds on the growing body of evidence suggesting that targeted VWM interventions may simultaneously enhance decoding fluency, reduce reading errors, and improve comprehension among students with SLD, offering a more comprehensive intervention framework.

Interventions to Improve Reading Fluency: To address fluency difficulties commonly experienced by students with SLD, evidence-based fluency interventions have been widely implemented in the literature (Therrien, 2004; Young et al., 2015). These approaches aim to strengthen word recognition automaticity, enabling students to read more quickly and accurately. Among the most effective strategies are repeated reading, where students practice reading the same text multiple times to improve both speed and accuracy, and timed reading exercises, designed to encourage students to gradually increase their reading rate (Hawkins et al., 2011; Lee & Yoon, 2017; Hudson et al., 2005). Modeling techniques, in which fluent reading is demonstrated by a skilled reader (e.g., teacher or parent), also play a critical role in fostering proper prosody and expression. These interventions provide structured, targeted practice opportunities, equipping students with essential fluency skills required for more efficient text processing and supporting overall reading development, particularly for students with persistent reading difficulties (Morgan & Sideridis, 2006; Wexler et al., 2008).

Interventions for Word Decoding and Reading Accuracy: In addition to fluency-focused strategies, interventions designed to enhance word decoding and reading accuracy also play a critical role in supporting students with SLD (Mayer & Motsch, 2015). These interventions primarily aim to strengthen phonological awareness, phonological coding, and morphological awareness, all of which are essential for accurate and efficient decoding. Phonological awareness training, including activities such as phoneme segmentation, blending, and deletion, helps students analyze word structures more effectively (Snowling & Hulme, 2011). Similarly, explicit letter-sound correspondence instruction enhances students’ ability to decode unfamiliar words, a skill particularly crucial for students with dyslexia or severe decoding deficits (Nunes et al., 2006). Morphological awareness training, which focuses on recognizing roots, prefixes, and suffixes, further supports accurate decoding and enhances vocabulary knowledge (Donegan & Wanzek, 2021; Stevens et al., 2017). By systematically strengthening these foundational skills, decoding interventions not only reduce reading errors but also contribute to more fluent and accurate reading, thereby indirectly supporting comprehension processes.

Cognitive Interventions: In addition to academic interventions targeting decoding and fluency, cognitive interventions have gained increasing attention in addressing the underlying cognitive deficits contributing to reading difficulties in students with SLD (Swanson & O’Connor, 2009). Among these, interventions focusing on VWM are particularly promising due to VWM’s central role in language processing and reading comprehension (Baddeley, 2000). VWM supports the temporary storage, processing, and integration of linguistic units such as words, phrases, and sentences during reading, enabling the construction of coherent mental representations essential for comprehension (Cain & Oakhill, 2006). While working memory consists of both verbal and visuo-spatial components, this study specifically focuses on VWM, given its direct relevance to language-based processing tasks involved in reading (Gathercole & Baddeley, 1993). In contrast, visuo-spatial working memory supports spatial organization and non-verbal reasoning, which are less directly involved in text comprehension. Previous research has consistently shown that deficits in VWM are strongly linked to reading comprehension difficulties, particularly in students with SLD, making it a critical target for intervention (Swanson & O’Connor, 2009; Cain & Oakhill, 2006). By isolating the role of VWM, this study aims to provide a clearer understanding of how targeted VWM training influences not only comprehension but also foundational reading processes such as reading speed and reading accuracy.

Working memory is a cognitive system responsible for the temporary storage and processing of information during complex cognitive tasks such as reading. It consists of two primary components: visuo-spatial working memory and VWM (Gathercole & Alloway, 2008). While visuo-spatial working memory supports spatial reasoning and visual organization, VWM plays a far more critical role in language processing and reading comprehension, as it enables the temporary retention, processing, and integration of linguistic units such as words and sentences (Gathercole & Alloway, 2008; Cowan, 2014).

Verbal Working Memory (VWM): VWM plays a critical role in the reading process by enabling the temporary storage, manipulation, and integration of linguistic information during reading (Carretti et al., 2009; Schwering & MacDonald, 2020). Through these mechanisms, VWM supports both word-level processing and higher-order comprehension, allowing readers to establish coherent meaning across sentences and paragraphs (Ober et al., 2019; Seigneuric & Ehrlich, 2005). However, research consistently indicates that students with SLD exhibit significant deficits in VWM, which disrupts their ability to retain and process linguistic information efficiently during reading (Swanson et al., 2009; Gathercole et al., 2006). These deficits hinder students’ ability to accurately recognize words, maintain sentence-level coherence, and integrate textual information into a meaningful whole. As a result, students with weak VWM often display reduced reading fluency, increased reading errors, and fragmented comprehension processes. Over time, these persistent difficulties contribute to broader academic underachievement, particularly in subjects that heavily rely on reading comprehension (Gathercole et al., 2006).

In response to these challenges, intervention studies targeting VWM have aimed to enhance reading performance by strengthening cognitive capacity for linguistic processing. Findings from these studies suggest that VWM training can yield moderate to significant improvements in reading comprehension (Dahlin, 2011). However, evidence regarding the effects of VWM interventions on reading fluency and reading accuracy remains inconsistent, with some studies reporting indirect gains (Melby-Lervåg & Hulme, 2013) while others found limited or no transfer effects to these foundational reading processes (Peng & Fuchs, 2016).

Given these inconclusive findings, further research is warranted to examine the comprehensive effects of VWM training on reading speed, reading errors, and comprehension performance in students with SLD. The current study directly addresses this gap by investigating whether targeted VWM intervention can simultaneously enhance VWM capacity, increase reading fluency, reduce decoding errors, and improve comprehension accuracy.

This study aims to examine the effects of VWM intervention on the VWM performance, reading speed, reading errors, and reading comprehension skills of students with SLD. The research seeks to explore how VWM training can improve the reading performance of these students and address the cognitive deficits in their reading processes.

To achieve this aim, the following hypotheses are posed:

**H1.**  *The VWM intervention will lead to a significant intra- and inter-group difference in the verbal working memory performance of the experimental group compared to the control group.*

**H2.**  *The VWM intervention package will significantly affect the reading speed (measured by the number of correctly read words per minute) of the experimental group compared to the control group, both within and between groups.*

**H3.**  *The VWM intervention package will result in a significant intra- and inter-group difference in the number of reading errors made by the experimental group compared to the control group.*

**H4.**  *The VWM intervention package will have a significant effect on the accuracy rate of reading comprehension in the experimental group compared to the control group, both within and between groups.*

## 2. Methods

### 2.1. Participants

The study employed a randomized pre-test and post-test control group experimental design to examine the effects of the VWM intervention. A total of 26 elementary school students officially diagnosed with SLD by the Ministry of Health were included in the study. Following participant selection, random allocation was conducted using a lottery method to assign students to either the experimental group (n = 14) or the control group (n = 12). The experimental group underwent a structured verbal working memory training program, while the control group continued their regular educ, ational activities without any specialized intervention. All participants were third- and fourth-grade students with an official SLD diagnosis. Information about the participants is presented in Table 1.

Due to the specific focus on students officially diagnosed with SLD, the pool of eligible participants was naturally limited. Additionally, logistical challenges, such as obtaining parental consent, securing school permissions, and coordinating the schedules of the students, further constrained the sample size. The individualized nature of the intervention, which required one-on-one or small-group sessions, also made it difficult to include a larger number of participants within the study timeline. While this small sample size may limit the statistical power and generalizability of the findings, effect size calculations (e.g., Cohen’s d) were used to strengthen the interpretation of the results.

### 2.2. Measurement Tools

In this study, the dependent variables, including the verbal memory subtests of the Working Memory Scale (WMS) and the reading tests from the SLD Clinical Observation Clinical Battery (SLD-COB), were utilized to examine the effects of the VWM intervention on the reading performance of students with SLD. The independent variable in this research is the VWM intervention. The following instruments were employed to collect data on the dependent variables.

Working Memory Scale (WMS): The WMS, specifically designed to assess the working memory performance of children aged 5 to 10, was utilized in this study (Ergül et al., 2018). The scale includes nine subtests across four dimensions: verbal/visual short-term memory (STM) and verbal/visual WM. In the verbal STM dimension, digit and word recall tasks are included, while pattern and block recall tasks are part of the visual STM dimension. The verbal WM dimension includes backward digit recall and first word recall, while the visual WM dimension comprises spatial recall and odd-one-out tasks. All tests are administered with increasing difficulty levels, and performance is scored based on the number of correct responses. In this study, the verbal STM subtests (digit and word recall) and verbal WM subtests (backward digit recall and first word recall) were employed. These subtests are structured such that higher scores indicate stronger working memory performance. The internal consistency, as measured by Cronbach’s Alpha, ranged from 0.68 to 0.99, confirming the reliability of these subtests.

SLD Clinical Observation Battery (SLD-COB): This battery, which was expanded by adding new subtests to the original SLD-COB developed by Korkmazlar (1992), includes nine subtests covering various cognitive and academic areas, such as mathematics, Gesell developmental assessments, writing, clock drawing, right-left discrimination, lateralization, and order of operations tests. Each subtest is interpreted as either supporting or refuting an SLD diagnosis. Reading speed was determined by calculating the ratio of words read per minute to the expected word count for the student’s age. This battery includes reading passages, a reading error form, and reading comprehension questions. Reading speed was measured by the number of words correctly read within one minute. Reading errors were defined as the number of mistakes made while reading the passage, and comprehension was assessed by the number of correct answers provided to questions related to the text (Karakaş et al., 2017; Turan et al., 2016).

### 2.3. Process

#### Verbal Working Memory Intervention

In this study, the VWM intervention was developed and implemented by the researchers. The intervention was specifically designed to strengthen students’ capacity to retain and process verbal information, focusing on both verbal short-term memory (V-STM) and VWM capacities. The intervention followed a structured three-stage process, with cognitive load systematically increasing at each stage, thereby progressively challenging the participants’ WM capabilities. The development of the intervention package involved contributions from two special education experts, ensuring that the activities were both pedagogically robust and tailored to meet the specific cognitive profiles and requirements of the participants. The 4-Week VWM Intervention was designed as a structured, group-based training aimed at enhancing students’ verbal working memory capacity and supporting its role in improving reading skills. The intervention was implemented over 4 weeks, comprising a total of 24 sessions, with 6 sessions per week. Each session lasted 25 min, resulting in an overall intervention duration of approximately 10 h.

The intervention was carefully structured into three stages, with progressively increasing cognitive demands. Throughout the process, diverse activities were implemented to target various aspects of verbal memory, combining explicit instruction with practice-oriented tasks. A central aim was to strengthen students’ capacity to store and process verbal information in both V-STM and VWM, promoting real-time processing and task execution.

The first week focused on acclimating students to the intervention process and building rapport with the instructor to foster engagement and motivation. This initial phase aimed to ensure that students felt comfortable with the structure of the sessions and were mentally prepared for the upcoming tasks. Establishing this connection was key to promoting active participation and maintaining focus throughout the program. During this week, participants were introduced to the fundamental concepts of V-STM and VWM. The role of verbal memory in learning was explicitly explained, with emphasis on how V-STM supports the temporary storage of information, while VWM enables real-time processing and task execution. Students were guided through examples illustrating how verbal memory impacts reading, comprehension, and academic performance. Key activities included the following: word and number repetition tasks to enhance short-term retention, word and sentence recall exercises to strengthen V-STM, and discussions highlighting the differences between V-STM and VWM and their relevance in daily tasks. Additionally, the online instruction process was introduced (if applicable), ensuring students understood the tools and expectations of the digital environment. This helped participants adapt to the structure and pace of the sessions. To establish a baseline, students completed short assessments evaluating their existing verbal memory skills. This allowed the instructor to tailor activities based on individual needs and set clear goals for the upcoming weeks.

The second week focused on teaching specific memory strategies aimed at strengthening verbal working memory. The goal was to help students not only retain verbal information more effectively but also improve their ability to organize and manipulate this information in real time. Participants were introduced to key strategies, including the following: Rehearsal Techniques: Students practiced mentally rehearsing verbal information through repetition-based tasks. These exercises aimed to improve information retention and recall efficiency. Sequencing Tasks: Activities involved organizing both meaningful and meaningless word sequences, strengthening students’ ability to maintain order and context in short-term memory. Phonological Loop Development: Exercises were designed to strengthen the phonological loop—a core component of VWM responsible for holding speech-based information. Students practiced retaining and processing verbal sequences through auditory repetition tasks. Throughout the week, participants received explicit instruction on how these strategies function and how they could be applied in academic settings, particularly in reading and comprehension tasks. By the end of the week, individual progress was assessed to determine students’ proficiency in using these memory strategies.

The fourth and final week focused on reinforcing learned strategies and promoting their application in diverse, real-life contexts. The primary aim was to help students internalize the strategies and develop the flexibility to adapt them across different tasks. Throughout the week, students engaged in activities that required the application of multiple strategies within varied scenarios, further strengthening their cognitive flexibility and problem-solving skills. Participants were encouraged to apply learned strategies in a range of tasks, promoting generalization beyond the intervention setting. Focus on improving fluency in strategy use, ensuring that techniques could be used effortlessly in academic and everyday contexts. Reflect on how the strategies could be adapted to future learning challenges. By the end of the week, participants had gained the tools needed to integrate these strategies into their academic work, particularly in reading comprehension and information processing tasks.

The intervention was developed in collaboration with two special education experts, ensuring its pedagogical soundness and relevance to students’ cognitive needs. Delivered in a group-based format, all participants received identical instruction and participated in the same activities to maintain consistency.

A session control checklist was used during each session to monitor key components, including entry routines, instructional processes, and closure routines. Implementation fidelity was evaluated by reviewing four randomly selected sessions, which confirmed an 88% fidelity rate. Sessions adhered to the planned structure and timing, ensuring consistency across all participants.

For interventions conducted in an online format (if applicable), special attention was given to the digital tools used and students’ adaptation to the virtual environment. This included guidance on using online platforms and strategies for maintaining engagement during remote sessions.

### 2.4. Data Collection and Analysis

Data were gathered individually using the WMS and SLD-COB reading tests. Upon completion of the intervention for the experimental group, post-test data were collected from both the experimental and control groups. To evaluate the data, the Shapiro–Wilk test was used to assess the normality of pre-test and post-test scores in verbal memory, reading speed, reading errors, and reading comprehension. Due to its suitability for small samples, the Shapiro–Wilk test provided a dependable measure of normality. For data that did not meet the assumption of normal distribution, the non-parametric Mann–Whitney U test was applied. In cases where normal distribution was confirmed, independent samples t-tests were used to evaluate differences between pre-test scores. Effect size was calculated using Cohen’s d and classified as follows: d ≈ 0.2 for a small effect, d ≈ 0.5 for a medium effect, and d ≥ 0.8 for a large effect (Cohen, 1988).

## 3. Results

### 3.1. Analysis of the Comparison of Reading Speed, Reading Errors, Reading Comprehension, and Verbal Memory Pre-Test Scores

The VWM, reading speed, reading errors, and reading comprehension pre-test scores of students in the experimental and control groups were compared to assess whether any statistically significant differences existed prior to the intervention. Analysis of the pre-test scores revealed no statistically significant differences in VWM performance (*t* = −0.52, *p* = 0.60, *p* > 0.05), suggesting that the groups were comparable at baseline. Similarly, no significant differences in reading speed were identified (U = 74.5, *p* = 0.64, *p* > 0.05), nor in the number of reading errors (*U* = 87.5, *p* = 0.87, *p* > 0.05). Finally, the reading comprehension test also indicated no significant group differences (*t* = −0.13, *p* = 0.90, *p* > 0.05). These findings confirm that the experimental and control groups were statistically similar across all measured variables prior to the intervention, ensuring a balanced comparison for subsequent analyses. The normality distribution data of the experimental and control groups, including the Shapiro–Wilk statistic values and *p*-values, are presented in Table 2.

The Shapiro–Wilk normality test results for the measurements from the experimental and control groups are presented in Table 2. According to the Shapiro–Wilk test, variables with a *p*-value greater than 0.05 were deemed to adhere to normal distribution assumptions, while those with a *p*-value less than 0.05 deviated from normality. In the experimental group, the variables Reading Speed Pre-Test, Reading Error Pre-Test, and Reading Comprehension Pre- and Post-Test deviated from normal distribution. Similarly, in the control group, the Reading Error Pre-Test variable also failed to meet the normality assumption. As a result, nonparametric tests were applied to these variables. Parametric tests were employed for all other variables that adhered to normality in both the experimental and control groups. The selection of parametric or nonparametric tests was contingent upon the underlying distribution properties of the variables.

### 3.2. Pre- and Post-Test Results of Reading Performance and Verbal Working Memory

This study systematically investigated the impact of a verbal working memory intervention on reading performance, utilizing a robust experimental design. The results detailing the effects of verbal memory on students’ reading performance are presented in Table 3.

To test Hypothesis 1 (H1), which posited that the VWM intervention would lead to a significant intra- and inter-group difference in the VWM performance of the experimental group compared to the control group, the following analyses were conducted. VWM findings: A statistically significant difference was identified between the pre-test mean (*M* = 13.50, *SD* = 1.70) and the post-test mean (*M* = 20.29, *SD* = 3.61) for the experimental group in VWM (*t*(13) = −7.78, *p* < 0.05), showing that the intervention had a substantial impact on VWM. In contrast, the control group’s pre-test mean (*M* = 14.00, *SD* = 3.05) and post-test mean (*M* = 15.00, *SD* = 3.53) revealed no statistically significant change *(t*(11) = −1.97, *p* = 0.074, *p* > 0.05), indicating the absence of notable improvement in VWM for the control group. A comparison of post-test scores between the experimental and control groups revealed a statistically significant difference (*t*(24) = 3.39, *p* < 0.05), indicating that the experimental group achieved significantly greater gains in VWM performance than the control group. Moreover, the effect size for the experimental group was calculated (d = 1.48), representing a large effect (d > 0.80). These results confirm that the intervention produced both statistically significant and practically meaningful enhancements in VWM, supporting H1.

To test Hypothesis 2 (H2), which proposed that the VWM intervention would significantly affect the reading speed (measured by the number of correctly read words per minute) of the experimental group compared to the control group, both within and between groups, the following analyses were conducted. Reading Speed Findings: The pre-test mean score for reading speed in the experimental group was *M* = 57.50 (*SD* = 16.11), and the post-test mean score was *M* = 62.86 (*SD* = 17.09). According to the Wilcoxon test results for the experimental group, no significant difference was found between the pre-test and post-test scores (*W* = 29, *p* = 0.15, *p* > 0.05). In the control group, the pre-test mean score for reading speed was *M* = 59.33 (*SD* = 25.26), and the post-test mean score was *M* = 63.33 (*SD* = 25.32). The difference in reading speed scores for the control group was analyzed using a t-test, which revealed no statistically significant difference (*t*(11) = −1.57, *p* = 0.15, *p* > 0.05). A comparison between the groups showed no statistically significant difference in reading speed between the experimental and control groups (*p* = 0.64, *p* > 0.05, *U* = 93.50). Additionally, the effect size was calculated as Cohen’s d = 0.12, indicating a small effect of the intervention on reading speed. In conclusion, although both groups showed an increase in reading speed, these increases were not statistically significant, and the intervention had no notable effect on reading speed. Therefore, H2 was not supported.

To test Hypothesis 3 (H3), which suggested that the VWM intervention would result in a significant intra- and inter-group difference in the number of reading errors made by the experimental group compared to the control group, the following analyses were conducted. Reading Error Subtest Findings: The pre-test mean score for reading errors in the experimental group was *M* = 12.43 (*SD* = 10.20), and the post-test mean score was *M* = 10.71 (*SD* = 6.31). According to the Wilcoxon test results, no statistically significant difference was found between the pre-test and post-test scores in the experimental group (*W* = 27.5, *p* = 0.36, *p* > 0.05). For the control group, the pre-test mean score for reading errors was M = 9.58 (*SD* = 5.96), and the post-test mean score was M = 10.67 (SD = 5.52). The Wilcoxon test results for the control group also indicated no statistically significant change (*W* = 23, *p* = 0.37, *p* > 0.05). A comparison between the groups showed no statistically significant difference in reading errors between the experimental and control groups (*p* = 0.98, *p* > 0.05, *t*(24) = 0.02). The effect size was calculated as Cohen’s d = 0.00, indicating that the intervention had no significant effect on reading errors. In conclusion, neither group showed a significant change in reading error scores, and the intervention did not have a notable impact on reading error performance. Therefore, H3 was not supported.

To test Hypothesis 4 (H4), which hypothesized that the VWM intervention would have a significant effect on the accuracy rate of reading comprehension in the experimental group compared to the control group, both within and between groups, the following analyses were conducted. Reading Comprehension Subtest Findings: The pre-test mean score for reading comprehension in the experimental group was *M* = 2.93 (*SD* = 1.27), and the post-test mean score was *M* = 4.07 (*SD* = 1.00). According to the Wilcoxon test results, a statistically significant difference was found between the pre-test and post-test scores in the experimental group (*W* = 0, *p* = 0.00, *p* < 0.05), indicating that the intervention had a significant effect on improving reading comprehension in the experimental group. In the control group, the pre-test mean score for reading comprehension was *M* = 3.00 (*SD* = 1.60), and the post-test mean score was *M* = 2.92 (*SD* = 1.51). The Wilcoxon test results showed no statistically significant difference between the pre-test and post-test scores in the control group (*W* = 2, *p* = 0.56, *p* > 0.05). A comparison between the groups revealed a statistically significant difference in reading comprehension between the experimental and control groups (*p* = 0.04, *p* < 0.05, *U* = 123.0). Additionally, the effect size was calculated as Cohen’s d = 0.92, indicating a strong effect of the intervention. In conclusion, the intervention significantly improved reading comprehension performance in the experimental group, and this improvement was statistically significant when compared to the control group, supporting H4.

## 4. Discussion

This study systematically evaluated the effects of a VWM intervention, which combined V-STM and verbal working memory training, on the reading skills and memory performance of students with SLD. Results demonstrated that VWM training led to statistically significant improvements in VWM capacity and reading comprehension performance. However, the training had no significant effect on reading speed and reading accuracy. These findings suggest that VWM enhancement supports higher-order comprehension processes but does not directly influence word decoding speed or reading fluency.

Although previous research highlights the potential of VWM interventions to enhance reading abilities among students with SLD, the magnitude of these effects varies depending on individual cognitive profiles and contextual factors (Alloway & Alloway, 2010; Holmes et al., 2009). In particular, students with lower initial VWM capacity have been shown to benefit more from targeted memory interventions, demonstrating greater improvements in comprehension performance compared to peers with higher baseline memory skills (Swanson & O’Connor, 2009). Moreover, socio-economic context has been identified as an important moderating variable, influencing both baseline VWM capacity and responsiveness to training programs (Mnisi, 2024). This suggests that VWM interventions should be designed with flexibility, ensuring they can accommodate diverse socio-economic backgrounds and individual cognitive needs. While this study did not directly investigate the impact of socio-cultural or economic factors, the findings align with the broader literature emphasizing that effective VWM interventions require careful adaptation to students’ specific cognitive profiles (Swanson & O’Connor, 2009). Future research would benefit from exploring how tailored cognitive interventions, sensitive to both cognitive baselines and environmental factors, could optimize reading outcomes for students with SLD. Theoretically, this study aligns with Baddeley’s Working Memory Model (Baddeley, 2000) and emphasizes the critical role of VWM in higher-order cognitive processes. The findings reveal that enhancing VWM capacity significantly impacts reading comprehension but has limited effects on reading speed and accuracy. This observation is consistent with findings discussed in the literature (Swanson & O’Connor, 2009) and reinforces the view that VWM primarily supports comprehension processes rather than lower-level reading skills.

In conclusion, VWM interventions can be effective in developing inclusive educational strategies. However, individual and contextual differences must be considered, and interventions should be tailored to align with students’ cognitive profiles.

Discussion on VWM: VWM is widely recognized as a pivotal factor influencing academic performance in individuals with SLD. In particular, the limited capacity of VWM in students with SLD has been consistently shown to contribute to deficiencies in language processing, reading comprehension, and mathematical skills (Gathercole & Alloway, 2008). For instance, a study demonstrated that individuals with SLD exhibited significantly lower VWM capacity compared to their typically developing peers, which adversely affected their reading and comprehension performance (Swanson et al., 2009). This highlights the crucial role that interventions aimed at enhancing VWM could play in improving the academic outcomes of these students. Similarly, strategic memory training targeting VWM enabled individuals with SLD to process and recall both verbal and visuospatial information more efficiently (Henry & Millar, 1993; Peijnenborgh et al., 2016). These findings are consistent with the significant improvements in VWM observed in the experimental group in this study, where the students in the experimental group scored markedly higher on VWM tests compared to the control group.

Recent studies have reinforced and expanded upon these findings. Cardoso et al. (2025) investigated the impact of a neuropsychopedagogical motor program on preschoolers’ executive functions and language skills. Their findings revealed that targeted motor activities not only strengthened VWM but also improved cognitive flexibility and language processing, leading to lasting academic benefits. Research highlights the impact of VWM interventions on cognitive and academic outcomes, particularly for students with learning difficulties. Studies show that targeted VWM training improves problem-solving skills and reading comprehension by enhancing memory capacity and processing efficiency (Swanson, 2011; Peng & Fuchs, 2016; Carretti et al., 2009). These findings suggest that structured verbal memory interventions can significantly support learning by strengthening language processing and problem-solving abilities.

The findings of this current study contribute to this growing body of evidence, reinforcing the effectiveness of VWM interventions in improving cognitive performance in students with SLD. Notably, the results align with recent research emphasizing the role of multisensory and movement-based strategies in strengthening working memory and related academic skills. This study extends prior research by confirming that VWM-focused interventions not only enhance memory performance but also yield measurable improvements in language and problem-solving abilities.

The importance of WM enhancement programs is highlighted, noting that such interventions not only improve language skills in students with SLD but also contribute to advances in other cognitive abilities, such as mathematical problem-solving and logical reasoning (Alloway et al., 2006). It was specifically observed that individuals with SLD derive greater benefits from these interventions compared to their typically developing peers, and enduring positive effects of VWM interventions on both reading and overall cognitive performance were documented (Titz & Karbach, 2014). Consistent with these findings, the present study demonstrates that the VWM intervention yielded a substantial effect size (*d* = 1.48) in the experimental group, with a significantly greater improvement compared to the control group. These findings highlight the effectiveness of interventions targeting VWM as a method for enhancing memory performance in students with SLD.

Discussion on Reading Speed: The literature provides substantial evidence indicating that interventions aimed at enhancing memory skills, particularly WM capacity, can lead to improvements in reading speed (Dahlin, 2011; Gathercole & Alloway, 2008; Loosli et al., 2012). Empirical studies involving student populations have demonstrated that programs targeting WM improvement can result in significant increases in reading speed. However, research indicates that the extent of these improvements may vary depending on factors such as age, individual learning profiles, and baseline reading abilities. This highlights the importance of accounting for individual differences when evaluating the effectiveness of such interventions (Wanzek et al., 2018). Recent research has further explored the nuanced impact of VWM interventions on reading speed. Klimovich and Richter (2024) emphasized that metacognitive training can significantly reduce “mindless reading”—a common issue where students mechanically read without comprehension. Their findings indicate that readers with lower baseline WM capacity experience more substantial improvements in reading speed when interventions focus on enhancing metacognitive strategies alongside WM training (Klimovich & Richter, 2024). In contrast, Cardoso et al. (2025) found that a neuropsychopedagogical motor program, designed to enhance executive functions and language skills, had limited direct effects on reading speed. Instead, improvements were more pronounced in reading accuracy and comprehension, suggesting that while WM capacity is a factor in reading fluency, motor and executive function integration may not consistently boost reading speed (Cardoso et al., 2025). Further complicating the picture, Sayekti et al. (2024) examined the role of motor skill training on critical reading abilities and found that while WM capacity improved post-intervention, these gains did not directly translate to faster reading. Instead, students demonstrated better attentional control and error detection, suggesting that improvements in WM may contribute more to reading quality rather than speed (Sayekti et al., 2024). These findings align with the results of the current study, where the VWM program did not produce a significant increase in reading speed. This outcome highlights the complexity of the relationship between WM and reading fluency.

This lack of significant improvement in reading speed may be due to several factors. First, while VWM supports comprehension by facilitating information integration, its direct influence on decoding speed and reading fluency remains limited (Gathercole & Baddeley, 1993). Additionally, the duration and structure of the intervention may not have been extensive enough to produce measurable changes in foundational reading skills, which often require more time-intensive fluency-based interventions (Torgesen et al., 2001). Individual differences, such as baseline memory capacity and cognitive flexibility, may have also moderated the impact of the intervention. Finally, the assessment tools used might have been more sensitive to capturing changes in comprehension rather than in reading speed or accuracy, potentially masking subtle improvements in these areas.

While WM interventions may bolster related cognitive domains—such as reading comprehension and attentional control—their direct impact on reading speed remains inconsistent. Individual differences, particularly baseline memory capacity and cognitive flexibility, appear to play a critical role in moderating these effects.

In a study, it was found that WM interventions not only enhanced the processing speed of complex reading materials but also contributed to more efficient organization of eye movements during reading (Loosli et al., 2012). Additionally, the effects of WM interventions on reading speed were more pronounced in children with lower baseline memory capacities (Ramezani et al., 2021). These findings suggest that individual differences in memory capacity are a critical factor in determining the success of interventions. Overall, WM interventions have been shown to significantly improve both reading fluency and comprehension in students struggling with both visual and verbal memory deficits (Novaes et al., 2019). On the other hand, several studies have found that memory interventions fail to produce significant improvements in reading speed. A meta-analysis and another study concluded that memory interventions did not significantly enhance reading speed or reading accuracy (Melby-Lervåg & Hulme, 2013; Banales et al., 2015). Similarly, a meta-analysis found no direct transfer effect of memory interventions on reading skills and reported that these interventions did not significantly improve reading speed or accuracy (Schwaighofer et al., 2015). These findings indicate that WM training may not consistently result in long-term improvements in reading abilities, and its effects on overall reading performance appear to be limited. A study investigated the impact of memory interventions on reading fluency in students with language-based SLD and found that memory training had a limited effect on reading accuracy and did not lead to significant improvements in reading speed (Sprenger-Charolles et al., 2013).

Similarly, commercially available WM training programs were examined and reported to be ineffective in improving reading speed or accuracy (Redick et al., 2013). It was also emphasized that the transfer effects of WM training are limited, and such interventions do not consistently enhance the various components of reading performance (Shipstead et al., 2012). These findings highlight the complexity of the relationship between WM interventions and reading performance, suggesting that additional factors (e.g., cognitive flexibility, phonological awareness, fluency strategies) may contribute to reading outcomes. However, the findings of our current study suggest that the VWM program failed to produce a significant increase in reading speed. This implies that the impact of interventions on reading speed may vary depending on factors such as individual differences and initial memory capacity. Although individual differences and initial memory capacity were not directly assessed in this study, prior research has shown that these factors can influence the responsiveness to working memory interventions. For example, Swanson and O’Connor (2009) and Alloway and Alloway (2010) demonstrated that students with lower baseline memory capacity tend to show greater improvements in reading fluency following cognitive interventions. This suggests that the variability in reading speed outcomes observed in the current study may, in part, reflect individual cognitive differences.

These results align with prior studies that have reported no significant improvements in reading speed following WM interventions.

Discussion on Reading Errors: Reading errors serve as critical indicators of overall reading proficiency. The development of V-STM and WM capacities can significantly contribute to the improvement of these indicators. Research indicates that WM capacity is essential for the temporary storage and processing of information during reading, and increasing this capacity may lead to improvements in reading accuracy and a reduction in reading errors (Swanson & Siegel, 2011; Soriano-Ferrer & Echegaray-Bengoa, 2014). This is particularly evident in individuals with reading difficulties such as dyslexia, where memory interventions have been shown to enhance reading accuracy and reduce phonological errors, thereby facilitating more accurate reading and better comprehension of the material (Swanson & O’Connor, 2009; Torgesen et al., 2001; Cain et al., 2004). The influence of improved memory capacity on reading accuracy becomes more pronounced when considering the different components of reading skills, such as decoding, comprehension, and speed (Peng et al., 2018). Moreover, cognitive processes like phonological awareness, linguistic knowledge, attention, and processing speed are thought to contribute to changes in reading speed and accuracy. Consequently, memory interventions may exert a positive influence on these components, ultimately enhancing reading accuracy.

However, contrary to these positive findings, some studies report that memory interventions fail to yield significant improvements in reading accuracy or the percentage of correct reading, or that their effects are limited (Melby-Lervåg & Hulme, 2013; Schwaighofer et al., 2015). A meta-analysis revealed that memory interventions did not demonstrate a direct transfer effect on reading skills and did not significantly improve reading speed or accuracy (Schwaighofer et al., 2015). Similarly, commercially available WM training programs were reported to be ineffective in increasing reading speed or accuracy (Redick et al., 2013). Although memory interventions can lead to some improvements in individual awareness and attention skills, these effects do not always translate into enhanced reading performance. These findings highlight the critical role of well-designed and carefully implemented intervention programs in improving their effectiveness. Similarly, it was emphasized that memory interventions did not directly enhance reading speed or accuracy, and that the transfer effects of these interventions were limited (Banales et al., 2015; Shipstead et al., 2012). These studies draw attention to the complexity of the relationship between memory training and reading performance, suggesting that other components of reading skills—such as phonological awareness, decoding, and visual perception—may also play a significant role in this process. In this context, studies that found no significant improvement in reading accuracy or reduction in reading errors following verbal memory interventions suggest that the effects of such interventions may vary depending on individual differences, baseline verbal memory capacity, and the type of intervention used.

The findings of the present study align with these conclusions; following the implementation of verbal memory interventions, no significant increase in reading accuracy or reduction in reading errors was observed. Several factors may explain the absence of significant improvements in reading accuracy and the reduction of reading errors observed in this study. First, while VWM plays a critical role in maintaining and processing linguistic information during reading, its direct influence on decoding accuracy and phonological processing is often limited (Swanson & O’Connor, 2009). Interventions targeting VWM primarily enhance higher-order cognitive functions, such as comprehension and information integration, rather than directly impacting lower-level processes like decoding and word recognition. Additionally, foundational reading skills such as phonological awareness and decoding accuracy often require specialized and fluency-focused interventions to yield measurable improvements (Torgesen et al., 2001). The relatively short duration of the current intervention may have further limited its capacity to impact reading accuracy. Moreover, individual differences among participants, including baseline reading skills and cognitive flexibility, could have moderated their responsiveness to the intervention. Lastly, the assessment tools employed may have been more sensitive to capturing comprehension gains rather than subtle shifts in reading accuracy, potentially underrepresenting minor improvements in this area.

These results suggest that the direct impact of VWM training on reading skills remains limited, highlighting the need for further investigation into complementary factors influencing reading performance.

Discussion on Reading Comprehension: The relationship between V-STM and WM interventions and reading comprehension skills is multifaceted. WM plays a pivotal role in comprehension, as individuals must temporarily retain and process the material they are reading. However, some studies suggest that the effects of these interventions on comprehension skills remain ambiguous or neutral. A systematic review indicated that VWM training generally holds potential to improve reading comprehension, yet this effect can exhibit variability across individuals and is not always statistically significant (Spencer-Smith & Klingberg, 2015). Similarly, a meta-analysis found that the impact of WM interventions on reading comprehension depends on factors such as the individual’s baseline memory capacity, age, and current reading proficiency (Peng et al., 2018). These findings highlight the significant variability in the effectiveness of WM interventions, which appears to be influenced by individual differences, learning difficulties, and the duration of educational programs. However, several studies also demonstrate the substantial impact of improving verbal memory skills on reading comprehension. It was suggested that enhancing WM skills has the potential to significantly boost reading comprehension abilities (Swanson, 2011). Similarly, it was demonstrated that verbal WM training led to substantial improvements in comprehension, particularly among students with SLD (Dahlin, 2011). The study found that children receiving memory training were able to maintain information for extended durations during reading, process it more efficiently, and establish relationships within the text more effectively. It was further identified that the link between WM and reading comprehension supports processes such as identifying main ideas and forming textual relationships (Artuso & Palladino, 2022). As such, interventions targeting the enhancement of verbal WM capacity can optimize comprehension by enhancing key cognitive processes involved in reading. Additionally, it was found that verbal WM training programs yielded measurable improvements across multiple components of reading proficiency, facilitating processes like identifying critical information, constructing context, and extracting overall meaning, thus contributing to enhanced comprehension outcomes (Fuchs et al., 2006).

A strong correlation between WM and reading comprehension was reported, highlighting that an increase in memory capacity can directly support comprehension (Cain et al., 2004; Schwering & MacDonald, 2020). In this study, verbal WM interventions administered to the experimental group resulted in a statistically significant enhancement in reading comprehension. Additionally, comparisons between the experimental and control groups revealed a meaningful difference in comprehension gains. These findings indicate that interventions targeting verbal WM can positively influence reading comprehension, leading to significant improvements in individuals’ reading performance. The reading comprehension literature consistently highlights the strong relationship between comprehension, reading fluency, and accuracy (Kim, 2020). However, in this study, although there were minor but statistically insignificant increases in reading speed and accuracy following the memory capacity intervention, a significant improvement in comprehension was observed. While this may appear contradictory at first glance, the substantial and significant effect size observed in the experimental group’s WM improvement, combined with their baseline reading speed and accuracy capacities, likely contributed to the notable advancement in comprehension. This suggests that participants in the experimental group were able to utilize their existing reading speed and accuracy more efficiently as a result of the improvements in WM. This finding aligns with and provides further evidence supporting prior research connecting WM and reading comprehension, as indicated in previous studies (Dahlin, 2011; Peng et al., 2018; Swanson, 2011).

This study’s intervention design offers several strengths that contribute to its practical and theoretical significance. First, the intervention directly targeted VWM through linguistically embedded tasks, ensuring task-specific training closely aligned with the target academic outcome (reading comprehension). This high degree of content relevance enhances the ecological validity of the training and maximizes its potential for near transfer, particularly for students with SLD, whose reading challenges are closely tied to verbal memory deficits (Swanson & O’Connor, 2009; Cain et al., 2004). Another key strength is the systematic training of both V-STM and VWM, recognizing that these two subsystems jointly contribute to successful reading performance. This dual focus ensured that students practiced not only simple retention skills but also the active manipulation and integration of verbal information, which are critical for higher-order comprehension processes (Baddeley, 2000; Gathercole & Alloway, 2008). However, the intervention also has several limitations that should be acknowledged. The absence of explicit fluency and decoding practice limited the intervention’s potential impact on reading speed and reading accuracy. As previous research highlights, automatic word recognition and efficient decoding processes depend heavily on direct fluency instruction, which cannot be fully compensated for through cognitive training alone (Torgesen et al., 2001; Hudson et al., 2005).

In addition, the group-based online delivery format, while practical, may have introduced variability in individual engagement and quality of practice, as students’ home environments were not controlled. Individual responsiveness to the intervention was not formally assessed, meaning that potential differential effects based on baseline VWM capacity remain unclear (Swanson & O’Connor, 2009). Overall, the intervention’s primary strength lies in its theoretically grounded, linguistically embedded focus on VWM, which aligns closely with the cognitive processes critical for reading comprehension. At the same time, its limited scope in directly addressing fluency and decoding processes highlights the importance of multicomponent intervention models that combine cognitive training with explicit reading instruction to comprehensively support reading development in students with SLD.

Although the findings of this study provide significant insights, there are several limitations to consider. The research was conducted over four weeks with 14 students in the experimental group and 12 in the control group, through a total of 26 online group sessions. Factors that may influence reading speed and reading accuracy (e.g., phonological awareness, attention skills) were not examined in detail. Additionally, the fact that the intervention was conducted online, the short duration of the program, and the inability to control whether the control group received any other special education support interventions are notable limitations of the study. Given the potential impact of these factors on the findings, the results should be interpreted with caution.

## 5. Recommendations

Practical Recommendations: It is recommended to integrate strategies for accurate and fluent reading with WM interventions. This approach should involve interventions applied over an extended period and tailored to individual needs. Such a combination will support the lasting development of reading skills, ensuring more sustainable improvements. Recommendations for Future Research: Longitudinal studies assessing the long-term effects of WM interventions on reading skills are suggested. Comparing different types of interventions is crucial to identify the most effective approach. Therefore, research that compares the effects of verbal working memory interventions and reading interventions, both when used together and separately in distinct experimental groups, should be conducted. These studies will provide a more comprehensive evaluation of the effectiveness of various intervention methods.

## 6. Conclusions

This study, conducted with students diagnosed with SLD, examined the effectiveness of VWM interventions and their relationship to reading performance. The findings demonstrated that VWM can be improved through targeted interventions and that these interventions can have a meaningful impact. However, the intervention did not result in statistically significant improvements in reading speed or accuracy. In contrast, notable improvements were observed in reading comprehension performance. Our results suggest that, while there may not be substantial gains in reading speed or accuracy, enhancing VWM capacity can significantly support reading comprehension skills. This finding highlights the importance of focusing on VWM interventions when the primary goal is to improve reading comprehension. It also suggests that educational interventions aimed at improving reading abilities, especially comprehension, should prioritize strategies that target WM. From an educational practice perspective, these results indicate that strategies designed to enhance VWM capacity can be effectively used to improve comprehension in students with SLD. In this respect, the study contributes to the literature on the relationship between reading skills and WM, providing important evidence that educational programs aimed at improving reading comprehension should incorporate WM interventions.

The findings of this study align with established cognitive models, such as Baddeley’s working memory model (Baddeley, 2000), which emphasize the pivotal role of VWM in supporting higher-order comprehension processes. While the intervention did not significantly impact reading speed or accuracy, the notable improvements in comprehension highlight VWM’s critical function in integrating and retaining information during reading. This distinction reinforces the theoretical understanding that VWM primarily aids complex cognitive tasks rather than lower-level processes like decoding. Additionally, the study highlights the importance of individualized intervention strategies for students with SLD, suggesting that cognitive profiles should be considered when designing reading interventions.

## Figures and Tables

**Table 1:** Descriptive characteristics of the participants.

Descriptive Feature of Groups		Experimental Groups		Control Groups		Total	
		*n*	%	*n*	%	*n*	%
Gender	Female	6	42.85%	5	41.66%	11	40.74%
	Male	8	57.14%	7	58.33%	15	59.25%
Grade Level	3rd Grade	11	78.57%	7	58.33%	18	69.23%
	4th Grade	3	21.42%	5	41.66%	8	30.76%
Total		14	53.84%	12	46.15%	26	100%
Test Mean		$\bar{X}$	SD	$\bar{X}$	SD		
Average Age		9.505	0.35	9.81	0.511		
Verbal Memory Pre-Test Scores		13.50	1.70	14.00	3.05		
Reading Speed Pre-Test Average (1 min)		57.50	16.11	59.33	25.26		
Reading Errors Pre-Test Count		12.43	10.20	9.58	5.96		
Reading Comprehension Pre-Test Accuracy Average		2.93	1.27	3.0	1.60		

**Table 2:** Normality distribution data for pre-test and post-test scores in the study.

Group	Statistical Terms	VWM Pre Test	VWM Post Test	Reading Speed Pre Test	Reading Speed Post Test	Reading Error Pre Test	Reading Error Post Test	Reading Compreh. Pre Test	Reading Compreh. Post Test
Exp.	*p*-value Wvalue	0.13 0.90	0.80 0.97	0.04 0.87	0.26 0.93	0.03 0.86	0.40 0.94	0.08 0.89	0.01 0.84
Control	*p*-value W value	0.64 0.95	0.96 0.97	0.34 0.93	0.45 0.94	0.04 0.85	0.60 0.95	0.16 0.90	0.16 0.95
Total	*p*-value Wvalue	0.47 0.96	0.59 0.97	0.90 0.98	0.68 0.97	0.00 0.85	0.64 0.97	0.01 0.90	0.00 0.88

**Table 3:** Reading performance findings of the experimental and control groups.

Measurement Area	Group	Pre-Test (M)	SS	Post-Test (M)	SS	Within-Group Difference (*p*) (*t*) (W)	Between-Group Difference (*p*) (*t*) (U)	Effect Size (d)
VWS	Exp. (*n* = 14)	13.50	1.70	20.29	3.61	*t* = −7.78 *p* = 0.00; *p* < 0.05	*t* = 3.39 *p* = 0.00; *p* < 0.05	d = 1.48
	Cont. (*n* = 12)	14.00	3.05	15.00	3.53	*t* = −1.97 *p* = 0.074; *p* > 0.05		
Reading Speed (1 min)	Exp. (*n* = 14)	57.50	16.11	62.86	17.09	W = 29 *p* = 0.15; *p* > 0.05	U = 93.50 *p* = 0.64; *p* > 0.05	d = 0.12
	Control (*n* = 12)	59.33	25.26	63.33	25.32	*t* = −1.57 *p* = 0.15; *p* > 0.05		
Reading Error	Exp. (*n* = 14)	12.43	10.20	10.71	6.31	W = 27.5 *p* = 0.36; *p* > 0.05	*t* = 0.02 *p* = 0.98; *p* > 0.05	d = 0.00
	Control (*n* = 12)	9.58	5.96	10.67	5.52	W = 23 *p* = 0.37; *p* > 0.05		
Reading Comprehension	Exp. (*n* = 14)	2.93	1.27	4.07	1.00	W = 0 *p* = 0.00; *p* < 0.05	U = 123.00 *p* = 0.04; *p* < 0.05	d = 0.92
	Control (*n* = 12)	3.00	1.60	2.92	1.51	W = 2 *p* = 0.56; *p* > 0.05		

VWM: verbal working memory; Exp: Experimental; Cont.: Control.

## Data Availability

The original data presented in the study are openly available in [FigShare] at [DOI/.10.6084/m9.figshare.27174636].
